# Immunocompromised individuals are at increased risk of COVID‐19 breakthrough infection, hospitalization, and death in the post‐vaccination era: A systematic review

**DOI:** 10.1002/iid3.1259

**Published:** 2024-04-25

**Authors:** Jola Bytyci, Yuxin Ying, Lennard Y. W. Lee

**Affiliations:** ^1^ Oxford Medical School University of Oxford Oxford UK; ^2^ Institute of Cancer and Genomic 22 Sciences University of Birmingham Birmingham UK; ^3^ Department of Oncology University of Oxford Oxford United Kingdom

**Keywords:** cancer, SARS‐CoV‐2, severe COVID‐19 outcomes, vaccine effectiveness

## Abstract

**Introduction:**

Immunocompromised individuals have been shown to mount a reduced response to vaccination, resulting in reduced vaccine effectiveness in this cohort. Therefore, in the postvaccination era, immunocompromised individuals remain at high risk of breakthrough infection and COVID‐19 related hospitalization and death, which persist despite vaccination efforts. There has been a marked paucity of systematic reviews evaluating existing data describing the clinical measures of efficacy of COVID‐19 vaccination, specifically in immunocompromised populations. In particular, there is a scarcity of comprehensive evaluations exploring breakthrough infections and severe COVID‐19 in this patient population.

**Methods:**

To address this gap, we conducted a systematic review which aimed to provide a summary of current clinical evidence of the effectiveness of COVID‐19 vaccination in the immunocompromised population. Using PRISMA guidelines, we conducted a literature search on PubMed and the Cochrane database published between January 1, 2021 to September 1, 2022.

**Results:**

Our findings demonstrated that despite vaccination, immunocompromised patients remained at high risk of new breakthrough COVID‐19 infection and severe COVID‐19 outcomes compared to the general population. We found increased average relative risk (RR) of breakthrough infections in the immunocompromised population, including patients with cancer (RR = 1.4), HIV (RR = 1.92), chronic kidney disease (RR = 2.26), immunodeficiency (RR = 2.55), and organ transplant recipients (RR = 6.94). These patients are also at greater risk for hospitalizations and death following COVID‐19 breakthrough infection. We found that the RR of hospitalization and death in Cancer patients was 1.08 and 2.82, respectively.

**Conclusion:**

This demonstrated that vaccination does not offer an adequate level of protection in these groups, necessitating further measures such as Evusheld and further boosters.

## Graphical abstract

We conducted a systematic review which aimed to provide a summary of current clinical evidence of the effectiveness of COVID‐19 vaccination in the immunocompromised population. Our findings demonstrated that immunocompromised patients remained at high risk of breakthrough infection and severe COVID‐19 outcomes compared with the general population. This demonstrated that vaccination does not offer an adequate level of protection in these groups, necessitating further measures such as Evusheld and further boosters.

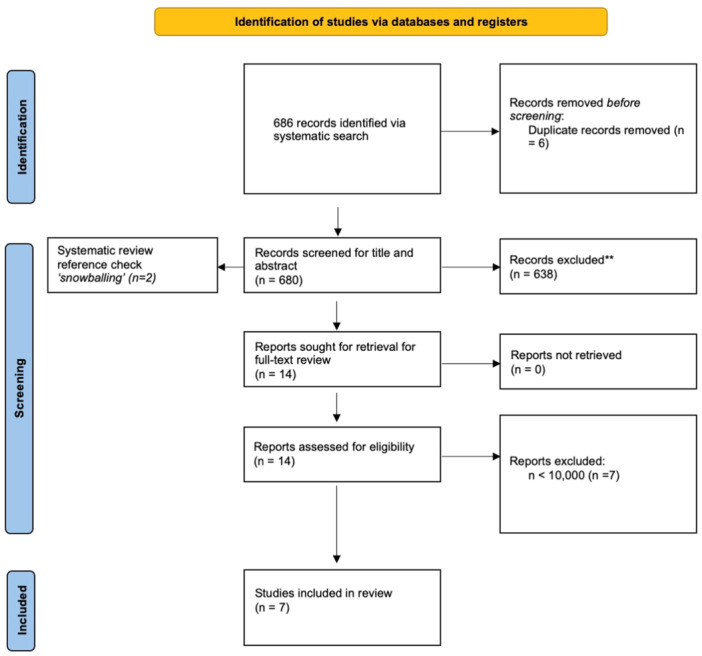

## 1 INTRODUCTION

Since the beginning of the COVID‐19 pandemic, immunocompromised (IC) individuals were identified as a high‐risk group due to an increased risk of severe COVID‐19 outcomes (hospitalization and death).^1^, ^2^, ^3^ IC individuals form a significant proportion of the population with around 500,000 individuals in the United Kingdom^4^ reported to be IC. In addition, most vaccine trials did not include an IC cohort.^5^ Thus, there was limited certainty that the outcomes of these trials would extend to the IC. Furthermore, this is supported by studies reporting weaker immunological responses to the vaccine in the IC cohort, including the OCTAVE study by NIHR, which investigated the humoral and T‐cell immune response to vaccination. At the time of its publication, the OCTAVE study was one of the biggest studies involving vaccinated IC individuals. They reported that 11% of IC patients did not generate any antibodies 4 weeks after two vaccines.^6^, ^7^, ^8^, ^9^ Moreover, the OCTAVE study reported that 40% of IC patients who seroconverted generated lower levels of SARS‐CoV‐2 antibody reactivity compared to non‐IC individuals postvaccination.^6^ To date, it has not been demonstrated that the third booster dose of the vaccine provides additional protection against severe COVID‐19 outcomes.^10^ The COVID‐19 vaccine offers protection against breakthrough infection (infection following a full course of vaccination) and reduces the incidence of severe COVID‐19 outcomes (hospitalization and death).^11^, ^12^ As IC patients mount a weaker immunological response to the vaccine, they are less protected against breakthrough infection, hospitalization, and death in the postvaccination era.^6^

This is heightened by the baseline increased risk that IC patients have to developing COVID‐19 infection and severe COVID‐19 outcomes.^13^, ^14^, ^15^, ^16^, ^17^, ^18^, ^19^ Additionally, between the COVID‐19 waves in England, the trends in risk reduction for IC patients did not follow that of the general population.^2^ Despite the relaxing of COVID‐19 public health measurers for the general public in the United Kingdom, the Office for National Statistics (ONS) UK estimates that at the end of December 2022, 1 in 20 people living in England were testing positive for COVID‐19.^20^ Given that research has shown that IC individuals are less likely to generate an effective vaccine response, the continuation of the pandemic and the potential surge in infections, these individuals remain at high risk of severe COVID‐19 outcomes.^6^, ^7^, ^8^, ^9^ Although the UK government is implementing the bivalent boosters, the fourth vaccine in the vaccination schedule, with the aim to further enhance protection against COVID‐19, it is still unclear whether this measure will protect IC individuals. There has been a marked paucity of systematic reviews evaluating existing data describing the clinical measures of efficacy of COVID‐19 vaccination in protecting IC individuals, specifically against, breakthrough infections and severe COVID‐19. Therefore, this systematic review aimed to provide a summary of current clinical evidence of the effectiveness of COVID‐19 vaccination in the IC population. This systematic review evaluated evidence from studies with a large N number and from multiple centers and will provide more robust evidence to current findings.

## 2 METHODS

This review is carried out following the Preferred Reporting Items for Systematic Reviews and Meta‐Analyses PRISMA guidelines^21^ and there were no significant deviations from the standard protocol.

### 2.1 Search strategy

A literature search was conducted on PubMed and the Cochrane database for studies conducted on humans and in the English language published between January 1, 2021 to September 1, 2022. The title and abstracts of studies were double‐screened by two reviewers independently. Our search terms across all databases were (COVID‐19 breakthrough infection OR breakthrough coronavirus infection OR covid‐19 related death OR covid‐19 related hospitalization) AND (cancer OR immunocompromised OR malignancy) AND (prospective OR cohort OR population‐based study).

### 2.2 Study selection

Primary outcomes of this review are to calculate the risk of breakthrough infections, COVID‐19‐associated hospitalizations and deaths in IC fully vaccinated individuals compared to non‐IC fully vaccinated individuals. Our inclusion criteria included: retrospective studies, observational studies, English, original findings, reports of breakthrough infections and/or COVID‐19 associated hospital admissions and deaths in fully vaccinated individuals. Our exclusion criteria included: study size less than 10,000 people, no or incomplete vaccination against COVID‐19, case reports, review articles, trial protocols, and nonoriginal findings. We excluded papers with a study size less than 10,000 people as we believe a larger sample size will be more representative of the general population. A comprehensive approach with taken with no limitations geographical location and population characteristics, not including COVID‐19 vaccination and IC status.

### 2.3 Data extraction

Reviewer Y. Y. and reviewer J. B. read selected papers independently and extracted the following data; year, study design, period, country, number of IC patients with two or more doses of COVID‐19 vaccinations, number of non‐IC patients with two or more doses of COVID‐19 vaccinations, number of COVID‐19 associated breakthrough infections, hospitalizations and deaths. We calculated the relative risk of breakthrough infections, hospitalizations and deaths by comparing the incidence of these outcomes in IC versus non‐IC populations. This enabled us to statistically characterize changes in the above parameters in the IC population versus non‐IC population.

In three instances we were not able to calculate the relative risk from the outlined method due to insufficient data. For Wang et al.,^22^ we extracted the hazard ratio for breakthrough infection in the cancer cohort. We calculated the ratio of overall risk for hospitalization and death of cancer patients compared with non‐cancer patients using fig. 3 from Wang et al.'s paper. For Agrawal et al.,^23^ we extracted adjusted rate ratios for COVID‐19‐related hospitalization or death from the supplementary appendix. In addition, we took the control population to be the 0 risk group cohort and the IC cohort with anyone that belonged to >/= 1 risk group. For Cox et al.,^24^ we extracted adjusted hazard ratios for hospitalization and death of IC groups directly from the paper. The data extracted were reviewed by Y. Y. and J. B., and any uncertainties in data extraction were discussed to ensure consistency and accuracy.

## 3 RESULTS

**Figure 1:**
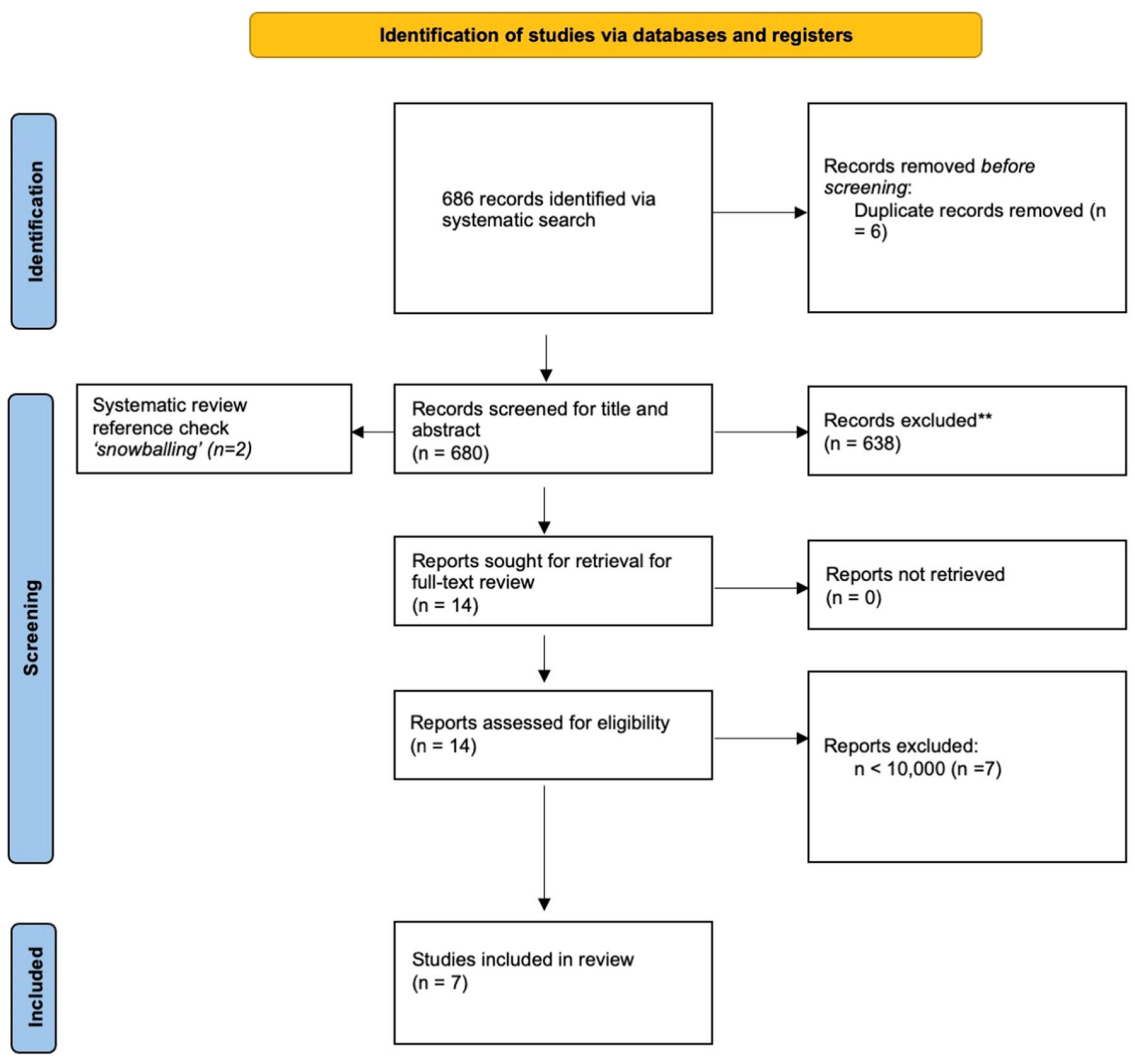
PRISMA flowchart.

Our literature search identified 686 records using our search terms. We removed 6 of these studies as they were duplicates. Applying our inclusion and exclusion criteria, 638 records were excluded following title and abstract screening and seven records were further excluded following full text review. Therefore, seven records were ultimately included. The complete search strategy is summarized in the PRISMA diagram (Figure 1). Our systematic review includes studies from the United Kingdom and the United States. The study period of data collection and further paper characteristics are summarized in Table 1.

**Table 1:** Included paper characteristics.

Authors	Title	Study design	Study period	Country	Primary outcomes	*N*	Vaccine type
Lee et al. (November 2022)^10^	COVID‐19: Third dose booster vaccine effectiveness against breakthrough coronavirus infection, hospitalizations and death in patients with cancer: a population‐based study	Population‐based test‐negative case‐control design	8/12/2020–7/12/2021	UK	Breakthrough infections, hospitalization and death	Cancer: 2258553 Control: 84,781,190	Pfizer BioNTech AstraZeneca
Lee et al. (May 2022)^25^	Vaccine effectiveness against COVID‐19 breakthrough infections in patients with cancer (UKCCEP): a population‐based test‐negative case‐control study	Population‐based test negative case control	8/12/2020–15/10/2021	UK	Breakthrough infections	Cancer: 377 194 Control: 28,010,955	Pfizer BioNTech AstraZeneca
Di Fusco et al.^26^	Evaluation of COVID‐19 vaccine breakthrough infections among immunocompromised patients fully vaccinated with BNT162b2	Retrospective	10/12/2020–8/07/2021	US	Breakthrough infections, hospitalization and death	IC: 225,796 Control: 1,051,951	Pfizer BioNTech
Wang et al.^22^	Breakthrough SARS‐CoV‐2 Infections, Hospitalizations, and Mortality in Vaccinated Patients With Cancer in the US Between December 2020 and November 2021	Retrospective cohort study	12/2020–11/2021	US	Breakthrough infection, hospitalization and death	Cancer: 45,253 Control: 591,212	Moderna Pfizer‐BioNTech Janssen/Johnson & Johnson
Liu et al.^27^	Risk Factors Associated With SARS‐CoV‐2 Breakthrough Infections in Fully mRNA‐Vaccinated Individuals: Retrospective Analysis	Observational retrospective analysis	01/2021–09/2021	US	Breakthrough infection	IC: 7746 Control: 9139	Pfizer‐BioNTech Moderna
Agrawal et al.^23^	Severe COVID‐19 outcomes after full vaccination of primary schedule and initial boosters: pooled analysis of national prospective cohort studies of 30 million individuals in England, Northern Ireland, Scotland, and Wales	Prospective cohort	08/12/2020–28/02/2022	UK	Severe outcomes (hospitalization and death)	IC: 7754880 Control: 8,416,050	Pfizer‐BioNTech AstraZeneca Moderna
Cox et al.^24^	Risk prediction of covid‐19 related death and hospital admission in adults after covid‐19 vaccination: national prospective cohort study	Prospective cohort	0/12/2020−15/06/2021	UK	Severe outcomes (hospitalization and death)	Total vaccinated cohort: 6,952,440	Pfizer‐BioNTech AstraZeneca

In evaluating who was most at risk following vaccination, the included papers looked at three main primary outcomes: breakthrough infections, hospitalizations, and death. The primary outcomes measured by each paper are summarized in Table 1.

Overall, IC patients displayed a trend of increased risk of breakthrough infection and COVID‐19‐related hospitalization and death. Our calculated relative risk for hospitalization for IC patients was 2.39 (*N* = 374), calculated from the patient cohort in Di Fusco et al.^26^ We found that the risk for breakthrough infection, COVID‐19‐related hospitalization and death differed between different immunocompromising conditions. Clinically, these findings suggest that further measures should be taken to reduce IC patient's risk from COVID‐19 infection and severe outcomes. This may involve prioritized access to diagnostics, preventative measures such as vaccination and early treatments.

### 3.1 Cancer

We identified five papers which a total *N* of X which reported on the breakthrough infections in vaccinated cancer cohorts versus vaccinated non‐cancer cohorts. The average calculated relative risk of breakthrough infections in cancer patient's versus non‐cancer patients was 1.4 (Table 2). The individual calculated relative risk from each paper ranged from 0.55 to 1.73, with only Lee et al.^25^ reporting data resulting in a calculated relative risk <1, 4 out of the five included papers independently showed a relative risk of >1.24.

**Table 2:** Calculated relative risk of breakthrough infections in the cancer cohort compared the non‐cancer cohort.

The relative risk of breakthrough infections in the cancer cohort compared with the non‐cancer cohort			
Authors	Vaccine type	*N*	RR
Lee et al. (November 2022)	Pfizer BioNTech AstraZeneca	2,258,553	1.73
Lee et al. (May 2022)	Pfizer BioNTech AstraZeneca	377,194	0.55
Di Fusco et al.	Pfizer BioNTech	81	1.84
Wang et al.^a^	Moderna Pfizer‐BioNTech Janssen/Johnson & Johnson	45,253	1.24
Liu et al.	Pfizer‐BioNTech Moderna	2400	1.62
Average			1.40

a Ratio of probability in patients with cancer versus non‐cancer patients.

**Table 3:** Calculated relative risk of breakthrough infections in non‐cancer immunocompromised cohort compared with non‐immunocompromised cohort.

The relative risk of breakthrough infections in non‐cancer immunocompromised cohort compared to non‐immunocompromised cohort						
Authors	Vaccine type	RR/N	Organ transplant	HIV	Chronic kidney disease	Immunodeficiency
Di Fusco et al.	Pfizer BioNTech	RR	11.62	2.28	2.95	3.50
		N	687	2103	41,597	3190
Liu et al.	Pfizer‐BioNTech Moderna	RR	2.25	1.56	1.56	1.60
		N	376	487	1514	2594
Average			6.94	1.92	2.26	2.55

*Note*: Patient definitions are available in the original papers, please refer for more information.

Lee et al. ^10^ and Wang et al.^22^ reported on hospitalizations and death in vaccinated cancer cohorts. The average calculated relative risk of COVID‐19‐related hospitalization in the cancer cohorts was 1.08 (0.92–1.23) whilst the average calculated relative risk of COVID‐19 death was 2.82 (2.43–3.21) (Table 4). Agrawal et al.^23^ reported adjusted rate ratios of severe COVID‐19 outcomes (defined as COVID‐19‐related hospitalization and death) in cancer cohorts. The adjusted rate ratio for blood and bone marrow cancers was 3.35 and for lung and oral cancers was 2.53 (Table 5).^23^

**Table 4:** Relative risk of hospitalization and death in cancer patients.

The relative risk of hospitalization and death in cancer patients				
Authors	Vaccine type	*N*	Hospitalization	Death
Lee et al. (November 2022)	Pfizer BioNTech AstraZeneca	2,258,553	0.92	3.21
Wang et al.^a^	Moderna Pfizer‐BioNTech Janssen/Johnson & Johnson	45,253	1.23	2.43
Average			1.08	2.82

^a^ Used ratio of probability in patients with cancer versus non‐cancer patients, immunocompromised group included only cancer patients. The ratio of probability was taken directly from the original paper due to a lack of data to calculate relative risk.

It is evident that despite a full vaccination course, cancer patients are not only at greater risk of COVID‐19 breakthrough infection, but also at a greater risk of hospitalization and death following these infections compared to the general population.

### 3.2 Transplants

We identified three papers with a total *N* of X which investigated outcomes in fully vaccinated transplant patients with Di Fusco et al.^26^ and Liu et al.^27^ reporting on breakthrough infections in transplant patients and Agrawal et al.^23^ reporting on severe COVID‐19 in transplant patients.

Overall, the calculated relative risk/reported adjusted rate ratios for transplant patients for breakthrough infection and severe COVID outcomes were significantly higher than 1 as summarized in Table 3. The relative risk of breakthrough infection for transplant patients were 11.62 and 2.25, giving an average relative risk of 6.94 (Table 3). Agrawal et al.^23^ reported that solid organ transplant recipients are at highest risk of severe COVID‐19 outcomes with an adjusted rate ratio of 23.35 (Table 5). This is followed by renal transplant patients with an adjusted rate ratio for severe COVID‐19 outcomes of 9.87 and by bone marrow transplant recipients with an adjusted rate ratio of 6.61.

**Table 5:** Adjusted rate ratios of hospitalizations and death in immunocompromised patients taken from Agrawal et al.

Condition	*N*	aRR
*Cancer*		
Blood/bone marrow	114,300	3.35
Lung/oral	42,560	2.53
*Transplants*		
Solid organ	11,130	23.35
Bone marrow	4750	6.61
Renal	1120	9.87
Immunosuppressants	188,310	5.8
*CKD*		
Stage 3	171,860	1.51
Stage 4	6910	3.22
Stage 5	7180	3.72
Chemotherapy	76,060	2.7

*Note*: Please refer to the original paper for definitions of the following IC conditions. aRRs taken directly from the original paper. Patient definitions are available in the original paper, please refer for more information.

Abbreviation: aRR, adjusted risk ratio.

Taken together, this highlights that transplant patients are at increased risk of breakthrough infection, hospitalization, and death from SARS‐CoV‐2 compared with the healthy population.

### 3.3 Chronic kidney disease

Several of the included papers reported on the susceptibility of chronic kidney disease patients to breakthrough infections and severe COVID‐19‐related outcomes. Generally, patients with chronic kidney disease had an average calculated risk of 2.26 (1.56–2.95) of COVID‐19 breakthrough infection, despite vaccination (Table 3). The vulnerability of these patients was further demonstrated in the findings of Agrawal et al.^23^ (Table 5) who reported adjusted risk ratios (aRR) of hospitalization and death of patients with chronic kidney disease. They reported aRRs of 1.51, 3.22, and 3.72 for stages 3, 4, and 5 of CKD, respectively (Table 5).

Supported by measures of both breakthrough infection and severe COVID‐19 outcomes, it is evident that patients with chronic kidney disease remain vulnerable in the postvaccination era.

### 3.4 Other conditions

We identified two studies which reported on breakthrough infection in fully vaccinated individuals with immunodeficiency and individuals with HIV as summarized in Table 3. Fully vaccinated individuals with immunodeficiency were at higher risk of breakthrough infection when compared to the healthy control population with an average relative risk of 2.55.

Similarly, patients with HIV were also at higher risk of breakthrough infection when compared to the healthy population with an average relative risk of 1.92. Not surprisingly, patients treated with immunosuppressants, and chemotherapy are at higher risk of severe COVID‐19 outcomes with adjusted rate ratios for hospitalization and death for immunosuppressants and chemotherapy reported at 5.8 and 2.7, respectively.

## 4 DISCUSSION

This systematic review assessed the existing data describing the efficacy of COVID‐19 vaccination in protecting IC individuals against breakthrough infections and severe COVID‐19 outcomes. These individuals are also more likely to experience severe complications from these breakthrough infections and they do not mount the same immune response following vaccination.^6^, ^7^, ^8^, ^9^ Whilst our results showed that IC patients are at increased risk of breakthrough infection, hospitalization and death (Tables 2, 3, 4, 5), this risk differed between different sub‐groups. Organ transplant patients were found to be the most vulnerable in this group with regard to breakthrough infection (RR = 6.94), then immunodeficient patients (RR = 2.55), followed by patients with chronic kidney disease (RR = 2.26), patients with HIV (RR = 1.92), and patients with cancer (average RR = 1.40) (Tables 2 and 3). Similarly, organ transplant patients were found to be the most vulnerable with regard to hospitalization and death postvaccination as this group demonstrated the highest adjusted rate ratios for hospitalization and death with solid organ transplants at highest risk (aRR = 23.35), followed by renal transplants (aRR = 9.87) and bone marrow transplants (aRR = 6.61) (Table 5). An increase in the risk of hospitalizations and death postvaccination can be observed with the increasing stages of CKD with stage 5 CKD (Table 5). The differences in relative risk between different sub‐groups of IC patients highlights important clinical considerations in the management of these patients. This may involve careful consideration regarding vaccination boosting strategies and public health interventions. Different cancer subtypes were found to carry different risks for breakthrough infection and severe COVID‐19 outcomes. Hematological cancer patients, particularly lymphoma, were consistently found to be a high‐risk group amongst included papers,^10^, ^22^, ^25^ with higher reported adjusted rate rations for severe COVID‐19 outcomes for blood and bone marrow cancer when compared to lung and oral cancer (Table 5). Interestingly, Liu et al.^27^ reported that cancer patients with a history of tumors did not exhibit a significant increase in risk suggesting that individuals whose cancers were in remission have a similar risk to the average population. With this, it seems that patients with active cancer and are undergoing active treatment are the most vulnerable.

Lee et al.^25^ reported that cancer patients had a reduced risk of breakthrough infection when compared to the general population. Whilst breakthrough infection is a metric of vaccine effectiveness, it carries more limitations than metrics such as COVID‐19‐related hospitalizations and death. Breakthrough infection reflects behavior as well as vaccine effectiveness, and, a shielded population (individuals who are clinically vulnerable and at high risk of severe COVID‐19 outcomes, for example, the IC cohort and are therefore isolating to reduce their risk of infection) may be less likely to encounter the virus. Therefore, this must be carefully considered when interpreting breakthrough infection as a metric for vaccine effectiveness. The waning immune response to the COVID‐19 vaccine has been well established in the population,^28^ and appears to occur at a more rapid rate in the IC population.^22^, ^25^ This is supported by work^23^ finding that there was an increased risk of severe COVID‐19 outcomes 10 weeks after primary vaccination.

Noting these risks, IC patients continued to maintain shielding behaviors to reduce their risks well into the postvaccination era. The final report on Coronavirus and clinically extremely vulnerable (CEV) people in England from the UK census stated that in April 2022 13% of people previously considered to be CEV to COVID‐19 continue to follow previous shielding advice and 69% continue to take extra precautions.^29^ In addition, 46% of CEV people reported feeling worried about the effect of COVID‐19 on their life compared with 34% of the general adult population in England.^29^ Clearly this requirement is likely to cause ongoing harms.

A limitation of our systematic review is that other immunocompromising conditions such as diabetes, which affect almost five million people in the United Kingdom, were not included.^30^ We found that diabetes was not a focus in our included papers. However, given the chronic nature of diabetes and its prevalence within the population, it is important not to ignore these conditions and thus more research is needed to explore vaccine effectiveness in this cohort.

Finally, at the beginning of the pandemic, certain demographic groups were a risk factor for infection and severe COVID‐19 outcomes. These included male sex, older age, certain ethnic groups, and low socioeconomic background amongst others.^31^, ^32^ As the pandemic has progressed with successive vaccine rollouts, there have been contradictory reports on whether these demographic factors remain risk factors for infection and severe COVID‐19 outcomes. Cox et al.^24^ reported that despite the vaccine rollout, certain ethnicities such as individuals from Indian and Pakistani ethnic origin, remained a risk factor for COVID‐19 mortality. However, Agrawal et al.^23^ disputed this and reported that the vaccine has diminished the difference in outcomes between different ethnic groups, whilst additionally identifying other demographic determinants of vaccine effectiveness including urban rather than rural settings. It is important to note that increased age has continually been identified as a risk factor for severe COVID‐19 outcomes.^10^, ^24^, ^26^ We recognize that a limitation of our systematic review is that our analysis did not account for age.

In summary, more action can be taken to protect IC individuals and support their reintegration into society. Additional booster vaccinations may be needed with associated research into the efficacy of boosters. In addition, risk stratification tools may be useful and necessary to identify those most at risk,^24^ specifically from our findings, this includes patients with cancer, chronic kidney disease, HIV, immunodeficiency, organ transplant recipients, patients undergoing chemotherapy and patients taking immunosuppressants. Further improvements to the immunogenicity of the vaccine have also been suggested,^23^ which could translate into improved vaccine efficacy in both the general and IC population. Additionally, ethical considerations must be recognized, including patient autonomy, non‐maleficence including an evaluation of side effects versus benefit of therapeutics and justice, such as the need to ensure fair access. Finally, innovative therapies, like long‐acting antibodies which provide alternatives to those who do not have immune responses to vaccines, such as Evusheld, sotrovimab or newer iterations could be made available to IC individuals as prophylaxis or treatment, particularly when infection rates are high.^33^, ^34^, ^35^, ^36^

## AUTHOR CONTRIBUTIONS

Yuxin Ying, Jola Bytyci, and Lennard Y. W. Lee designed the systematic review. Yuxin Ying and Jola Bytyci conducted the electronic search and reviewed publications for inclusion in the systematic review. Yuxin Ying and Jola Bytyci performed the data analysis. Yuxin Ying, Jola Bytyci, and Lennard Y. W. Lee drafted the manuscript. Jola Bytyci and Yuxin Ying are co‐first authors. Jola Bytyci and Yuxin Ying contributed equally to this systematic review. All authors have approved the manuscript for submission.

## CONFLICT OF INTEREST STATEMENT

The authors declare no conflict of interest.

## Data Availability

The data that support the findings of this study are available from the corresponding author upon reasonable request.
